# Association of rs662799 variant and *APOA5* gene haplotypes with metabolic syndrome and its components: a meta-analysis in North Africa

**DOI:** 10.1042/BSR20200706

**Published:** 2020-08-13

**Authors:** Meriem Hechmi, Hamza Dallali, Meriem Gharbi, Haifa Jmel, Meriem Fassatoui, Yossra Ben Halima, Sonia Bahri, Afaf Bahlous, Abdelmajid Abid, Henda Jamoussi, Abdelhamid Barakat, Rym Kefi

**Affiliations:** 1 Laboratory of Biomedical Genomics and Oncogenetics, Institut Pasteur de Tunis, BP 74, 13 Place Pasteur, Tunis 1002, Tunisia; 2 University of Carthage, National Institute of Applied Science and Technology, Tunis, Tunisia; 3 Institut Pasteur de Tunis, University of Tunis El Manar, 2092 El Manar I Tunis, Tunisia; 4 Central Laboratory of Medical Biology, Institut Pasteur de Tunis, BP 74, 13 Place Pasteur, Tunis 1002, Tunisia; 5 National Institute of Nutrition and Food Technology, 11 rue Jebel Lakhdar, Bab Saadoun, Tunis 1007, Tunisia; 6 Laboratory of Human Molecular Genetics Institut Pasteur du Maroc 1, Place Louis Pasteur, Casablanca, Morocco

**Keywords:** APOA5 Gene, genetic association, Metabolic Syndrome, North Africa, polymorphisms, Tunisia

## Abstract

Apolipoprotein A5 (*APOA5*) has been linked to metabolic syndrome (MetS) in several populations. In North Africa, only the Tunisian and Moroccan populations were investigated. Our aim is to assess the association between *APOA5* gene variant (rs662799) and haplotypes with MetS in Tunisian population and to perform a meta-analysis in North Africa. A total of 594 Tunisian participants were genotyped for polymorphism rs662799 using KASPar technology. Two polymorphisms rs3135506 and rs651821 in *APOA5* gene genotyped in our previous study, were used in addition to rs662799 to assess the haplotype association with MetS. The genotype of 875 participants was used for the meta-analysis. Statistical analyses were performed with R software.

The rs662799 increases the risk of MetS under the dominant (*P*=0.018) and the additive models (*P*=0.028) in the Tunisian population. After stratification of the cohort following the sex and the geographic origin, a positive association of rs662799 with MetS was found for participant from the Northern region and for the women group. Only the haplotype AGT showed a significant association with MetS by decreasing the risk of the disease.

The meta-analysis reported a significant association of rs662799 and rs3135506 with MetS.

Our results showed a significant association between the *APOA5* gene variants rs662799 and haplotypes with MetS and its traits in Tunisia. An impact of the sex and the geographic origin on the genotype distribution was highlighted. Our funding emphasizes the role of *APOA5* in the development of MetS in North Africa.

## Introduction

Metabolic syndrome (MetS) consists of a constellation of several metabolic abnormalities, including hypertension, dyslipidemia, abdominal obesity and insulin-resistance [1,2]. The prevalence of MetS is 25% in the world [3] and 30% in Tunisia [4]. This chronic disease can evolve to serious complications such as type 2 diabetes and cardiovascular diseases increasing the morbidity and the mortality of MetS patients [5]. Since MetS is a multifactorial disease, its etiology is still unclear. However, two main factors are the origin of MetS; the genetic background and the environmental factors. An unhealthy diet and sedentary lifestyle lead to the development of MetS added to the genetic susceptibility [6,7]. To date, studies have identified several SNPs to be correlated with MetS and its complications through genome-wide association studies (GWASs) and candidate gene association studies. Nevertheless, there is some inconsistency in the results of different studies.

Apolipoprotein A5 (*APOA5*) gene is mapped to the chromosome 11q23.3 in the *APOA1/C3/A4/A5* gene cluster, enclosing 4 exons coding for a 366-amino acid protein, APOAV. This protein has a key role in the lipid metabolism mainly triglyceride (TG) levels. APOAV is synthesized in the liver where it was identified for the first time. Although, the regulation mechanism of APOAV is not completely decoded, studies showed that after a liver injury an increase in the APOAV levels is observed. The synthesis of APOAV decreases the production of very low-density lipoprotein-TG (VLDL-TG) and at the same time enhances the activity of lipoprotein lipase (LPL) up to 2.3-fold which mediates TG hydrolysis [8,9]. *In vivo* and *in vitro* studies have deciphered a new function of the APOAV protein, which might be secreted in other tissues like intestine or the adipose tissue [10].

Several studies have identified *APOA5* gene to be strongly implicated in the establishment of cardiovascular diseases [11–14], impaired lipid traits, DT2 and MetS for different ethnic populations [15–18]. In our previous study, we reported the association of *APOA5* variants (rs651821 and rs3135506) with MetS and its traits [19]. Other studies reported the involvement of rs662799 (−1131T>C) in MetS [15,20,21].

The aim of the present work is to assess the association between *APOA5* gene variant (rs662799) and haplotypes with MetS in Tunisian population and to perform a meta-analysis in North Africa.

## Materials and methods

### Study subjects

The 594 Tunisian participants were recruited from the National Institute of Nutrition and Food Technology (Tunis, Tunisia) and Institut Pasteur in Tunis (IPT). Informed written consents were obtained from all subjects. A total of 299 controls and 295 MetS patients were involved in the present study. The ethical committee of IPT (IRB00005445, FWA00010074) reviewed and approved the present study (Reference IPT/LR11-05/Etude 04/2013) according to the principles of the Declaration of Helsinki.

MetS was diagnosed according to the International Federation of Diabetes (IDF). As a central criteria for MetS patients, they must have abdominal obesity (waist circumference ≥ 94 cm for men and ≥ 80 cm for women) added to two metabolic abnormalities among this list: (1) elevated blood pressure (systolic blood pressure (SBP) ≥130 mmHg or diastolic blood pressure (DBP) ≥ 85 mmHg) or treatment for hypertension; (2) low plasma HDL level (>1.03 mmol/l in men and >1.29 mmol/l in women) or specific treatment for lipid abnormality; (3) elevated plasma TGs (≥1.7 mmol/l) or specific treatment for lipid abnormality; (4) impaired fasting plasma glucose (IFG) ≥ 5.6 mmol/l or diagnosed with type 2 diabetes.

### Clinical features

All measurements were performed at the National Institute of Nutrition and Food Technology by medical doctors. Anthropometric parameters were measured for all participants for height, weight and the waist circumference. Body mass index (BMI) was assessed as weight (kg)/ height^2^ (m^2^). Total cholesterol (TC), HDL-cholesterol, LDL-cholesterol, TG and impaired fasting glucose (IFG) were measured using standard technics in the laboratory of biochemistry at IPT and NIN. LDL-c was assessed using the Friedewald equation. SBP and DBP were measured by the auscultatory method using a stethoscope and a sphygmomanometer.

### Genetic analysis

DNA was isolated from the whole blood using the salting out method [22]. Genotyping of SNPs was performed by KASPar® technology (*KBioscience, U.K.*). Genotyping was performed using the LightCycler 480® system (*Roche Diagnostics, Switzerland*). The genotyping success rate was 97.14%. A random of 10% sample set was re-tested with the same method to confirm genotype accuracy.

### Statistical analysis

R software was used to assess all the statistical analyses. Biochemical and clinical data were presented as mean ± SD, Hardy–Weinberg equilibrium (HWE) was calculated using the R package SNPassoc [23]. The comparison of the numerical variables between groups was carried out with Student’s *t* test. Regarding the categorical values, the comparison of the distribution between controls and cases was carried out using the chi-squared (χ^2^) test. The genotypic and allelic frequencies were computed using the compare Groups R package [24]. The correlations with MetS were computed using multivariate logistic regression model after adjusting for sex, age and BMI. Univariate and multivariate methods based on logistic regression analysis for dominant, recessive and additive models of inheritance were used in order to pinpoint the genetic effect of the single nucleotide polymorphisms (SNPs) and compare the genotype frequencies between cases and controls. The correlations between *APOA5* SNPs and MetS were presented as *P*-value (P), odds ratio (OR) and confidence interval (CI). ANOVA was applied to compute the genotype–phenotype association and interactions.

Linkage disequilibrium statistics were computed using D′ and r² tested with Haploview software (version 4.2) [25]. Haplotype frequencies and associations with MetS were estimated using PLINK software (version 1.07). A value of *P*<0.05 was considered statistically significant for statistical tests. As for the HWE, the *P*-value was set among controls at (α = 10^−3^) [26].

### Meta-analysis

Data of *APOA5* variants in North African populations were collected from literature by searching Pubmed using key words: *APOA5*, North Africa, rs662799 and rs3135506. We performed an allelic meta-analysis. Statistical heterogeneity was assessed using a chi-square-based *Q* test and quantified using *I^2^*. Mantel–Haenszel (fixed-effects) method was used when *P_Heterogeneity_*>0.1 or *I^2^* < 50%. Alternatively, the model of DerSimonian and Laird (random-effects) was used.

## Results

### Characteristics of the studied population

Biochemical and clinical features of the studied population (MeS patients and controls) are reported in Table 1. Our results showed significant differences of several features; high WC (waist circumference), BMI (body mass index), IFG (impaired fasting plasma glucose), TG (triglyceride), DBP (diastolic blood pressure), SBP (systolic blood pressure), LDL (low-density lipoprotein) and low level of HDL (high-density lipoprotein) in MetS patients compared with controls (*P*<10^−4^). Age was significantly higher in MetS patients (56.58 ± 8.56) than in controls (52.56 ± 10.09), *P*<0.001. Only TC did not show a significant difference between the two groups.

**Table 1:** Characteristics of the study population

MetS components	Control group (*n*=299)	MetS group (*n*=295)	*P*-value
Age (years)	52.56 ± 10.09	56.4 ± 8.50	**<0.001**
WC (cm)	97.08 ± 11.87	106.50 ± 9.90	**<0.001**
BMI (kg/m^2^)	28.41 ± 4.83	31.54 ± 5.11	**<0.001**
HDL (mM)	1.48 ± 0.41	1.13 ± 0.34	**<0.001**
LDL (mM)	3.12 ± 0.89	3.40 ± 1.05	**0.001**
DBP (cmHg)	7.74 ± 1.26	8.34 ± 1.40	**<0.001**
SBP (cmHg)	13.20 ± 1.97	14.53 ± 2.20	**<0.001**
FPG (mM)	6.13 ± 2.51	9.50 ± 4.25	**<0.001**
TC (mM)	5.09 ± 0.92	5.16 ± 1.01	0.372
TG (mM)	1.29 ± 0.55	2.02 ± 0.92	**<0.001**

Abbreviation: *n*, number. Data are presented as means ± standard deviation. Significant *P*-values (<0.05) are in bold.

### Association with MetS

The genotypic and allelic distributions of the SNP rs662799 in the studied population are reported in Table 2. The difference in the distribution of the risk allele between patients and controls is statistically significant (13.9 and 9.6%, respectively; *P*=0.019). This distribution obeys the HWE (*P*<0.05).

**Table 2:** Association of rs662799 genotypes with MetS in the Tunisian population

Genotype distribution					Additive model		Dominant model		Recessive model	
	MetS	Controls	OR (95% CI)	*P*	OR (95% CI)	*P*_adj_	OR (95% CI)	*P*_adj_	OR (95% CI)	*P*_adj_
rs662799										
AA	221 (75.2%)	248 (83.2%)	0.41 (0.61–0.91)	0.016*						
AG	64 (21.8%)	43 (14.4%)	1.65 (1.08–2.53)	0.021*						
GG	9 (3.1%)	7 (2.3%)	1.31 (0.48–3.57)	0.59	1.55 (1.04–2.31)	0.028*	1.77 (1.10–2.86)	0.018*	1.50 (0.47–4.75)	0.49
RAF	13.9%	9.6%	1.53 (1.07–2.19)	0.019*						
HWE	0.14	0.01								
AIC						661.6		660.8		665.9

Genotype distributions are shown as number (%). Abbreviations: AIC, Akaike Information Criterion; HWE, *P*-value for Hardy–Weinberg equilibrium; *P*_adj_, *P*-value adjusted for age, sex and BMI; RAF, risk allele frequency. *: Significant *P*-value (*P*-value <0.05).

The carriers of the G allele have an increased risk of developing MetS (OR = 1.53 (1.07–2.19); *P*=0.019). The distribution of the AA genotype is significantly higher in the control group (83.2%) compared with the patient group (75.2%); *P*=0.016. In addition, a significant difference is observed in the distribution of the AG genotype between controls (14.4%) and MetS (21.8%); *P*=0.021. The SNP rs662799 is significantly associated with MetS under the dominant model OR = 1.77 (1.10–2.86); *P*=0.018 and the additive model OR = 1.55 (1.04–2.31); *P*=0.028 after adjusting for sex, age and BMI. An association analysis was also performed after stratification of the studied population according to the sex (Table 3). Our results showed a strong association of the rs662799 with MetS in the women group under the additive model OR = 1.60 (1.02–2.52); *P*=0.037 and the dominant model OR = 2 (1.13–3.54); *P*=0.016. No correlation was found in the male group (Table 3).

**Table 3:** Genotypic distribution of *APOA5* variant in the studied population stratified following the sex

Women										
Genotype distribution					Additive model		Dominant model		Recessive model	
	MetS	Controls	OR (95% CI)	*P*	OR (95% CI)	*P*_adj_	OR (95% CI)	*P*_adj_	OR (95% CI)	*P*_adj_
rs662799										
AA	145 (75.5%)	182 (83.5%)	0.61 (0.38–0.99)	0.04*						
AG	40 (20.8%)	29 (13.3%)	1.72 (1.02–2.9)	0.043*						
GG	7 (3.6%)	7 (3.2%)	1.14 (0.39–3.31)	0.81	1.60 (1.02–2.52)	0.037*	2.00 (1.13–3.54)	0.016*	1.34 (0.41–4.39)	0.62
RAF	14.1%	9.9%	1.5 (0.98–2.3)	0.06						
HWE	0.09	0.001								
AIC						463.1		461.7		467.2

Men										
Genotype Distribution					Additive model		Dominant Model		Recessive model	
	MetS	controls	OR(95% CI)	P	OR(95% CI)	Padj	OR(95% CI)	Padj	OR(95% CI)	Padj
rs662799										
AA	74(77.9%)	66 (82.5%)	0.75(0.35-1.59)	0.44						
AG	20(21.1%)	14(17.5%)	1.26(0.59-2.69)	0.55						
GG	1(1.1%)	0	2.55(0.1-63.6)	0.56	1.47(0.62-3.48)	0.37	1.41(0.57-3.47)	0.45	−	0.31
RAF	11.6%	8.8%	1.37(0.68-2.77)	0.38						
HWE	0.840	0.803								
AIC						199.9		200.1		199.7

Hardy_Weinberg equilibrium. *: Significant p-value. Genotype distributions are shown as number (%). RAF: risk allele frequency. HWE: p-value for (p-value<0.05), AIC: Akaike information criterion. P*: p-value after Bonferroni correction. Padj: p-value adjusted for age, sex and BMI.

### Impact of the geographic origin

Since the Tunisian population displays a mosaic genetic background due to several successive civilizations and migratory flows [27,28] we investigated the effect of the geographic origin on the genotype distribution of the rs662799 (Table 4). A stratification of the cohort into Northern and Southern regions was performed before the statistical analysis. Our results highlighted a significant association of the rs662799 with MetS among the Northern region only. This association was observed under the additive model (OR = 1.65 (1.06-2.56); p = 0.024) and the dominant model (OR = 1.92 (1.13-3.26); p = 0.014) (Table 4).

**Table 4:** Genotypic distribution of *APOA5* variant in the studied population stratified following the geographic origin

Northern population								
Genotype Distribution			Additive model		Dominant Model		Recessive model	
	MetS	controls	OR(95% CI)	Padj	OR(95% CI)	Padj	OR(95% CI)	Padj
rs662799								
AA	184(76.3%)	188(85.1%)	1.65(1.06-2.56)	0.024^*^	1.92(1.13-3.26)	0.014^*^	1.52(0.42-5.47)	0.51
AG	50(20.7%)	28(12.7%)						
GG	7(2.9%)	5(2.3%)						

Southern population								
Genotype Distribution			Additive model		Dominant Model		Recessive model	
	MetS	controls	OR(95% CI)	Padj	OR(95% CI)	Padj	OR(95% CI)	Padj
rs662799								
AA	18(58.1%)	39(72.2%)	0.75(0.21-2.68)	0.65	0.95(0.22-4.16)	0.95	−	0.15
AG	12(38.7%)	13(24.1%)						
GG	1(3.2%)	2(3.7%)						

Genotype distributions are shown as number (%). *: Significant p-value (p-value<0.05)Padj: p-value adjusted for age, sex and BMI.

### Association with quantitative traits

The association analysis of the variant rs662799 with various quantitative traits such as IFG, LDL, HDL, TG, PAS and PAD under the dominant model is reported in Table 5. In order to choose the appropriate model, we assessed the Akaike Information Criterion (AIC). We picked the dominant model since it had the lowest AIC value. No significant associations were observed between this variant and none of the biochemical clinical traits nor blood pressure. Even after stratifying the population according to the sex no significant association was observed. Since rs662799 is known to be associated with cardiac problems, we performed an association with cardiovascular complications. No significant association was found (data not shown).

**Table 5:** Effect of rs662799 variant in *APOA5* Gene on MetS-related phenotypes stratified in male and female subjects

Traits	Total			Women			Men		
	GG+GA	AA	P*	GG+GA	AA	P*	GG+GA	AA	P*
WC(cm)	102.46±11.51	101.51±12.05	0.47	103.25±12.05	101.46±12.31	0.28	100.70±10.13	101.63±11.43	0.68
BMI(Kg/cm²)	30.11±5.26	30.04±5.21	0.90	30.82±5.29	30.98±5.35	0.81	28.40±4.86	27.82±4.09	0.49
IFG(mmol/l)	8.36±4.33	7.80±3.84	0.19	8.41±4.50	7.58±3.60	0.09	8.24±3.94	8.34±4.31	0.90
SBP(mmHg)	14.27±2.23	13.91±2.13	0.14	14.29±2.43	13.79±2.10	0.08	14.21±1.70	14.20±2.20	0.98
DBP(mmHg)	8.06±1.28	8.09±1.40	0.87	8.07±1.22	8.02±1.35	0.77	8.05±1.43	8.26±1.49	0.49
TC(mmol/l)	5.10±1.06	5.13±0.95	0.77	5.19±1.13	5.18±0.97	0.93	4.86±0.84	5.00±0.88	0.45
HDL(mmol/l)	1.32±0.45	1.31±0.41	0.85	1.42±0.47	1.37±0.39	0.36	1.07±0.26	1.17±0.40	0.19
LDL(mmol/l)	3.10±1.10	3.14±1.14	0.72	3.00±1.16	3.16±1.21	0.34	3.33±0.93	3.11±0.98	0.28
TG(mmol/l)	1.71±0.80	1.66±0.87	0.60	1.61±0.78	1.56±0.76	0.65	1.95±0.80	1.88±1.05	0.75

Data are presented as mean ± Standard Deviation. Student test was used to compare geometric mean levels of continuous characteristics across genotypes. WC, Waist circumference, SBP, systolic blood pressure; DBP, diastolic blood pressure; BMI, body mass index; TC, total cholesterol; TG, triglycerides; HDL-C, high density lipoprotein cholesterol; LDL-C, low density lipoprotein cholesterol; IFG, impaired fasting glucose. Significant results are in bold. P*: p-value adjusted by age, sexe and BMI

### Haplotype association and linkage disequilibrium

**Figure 1:**
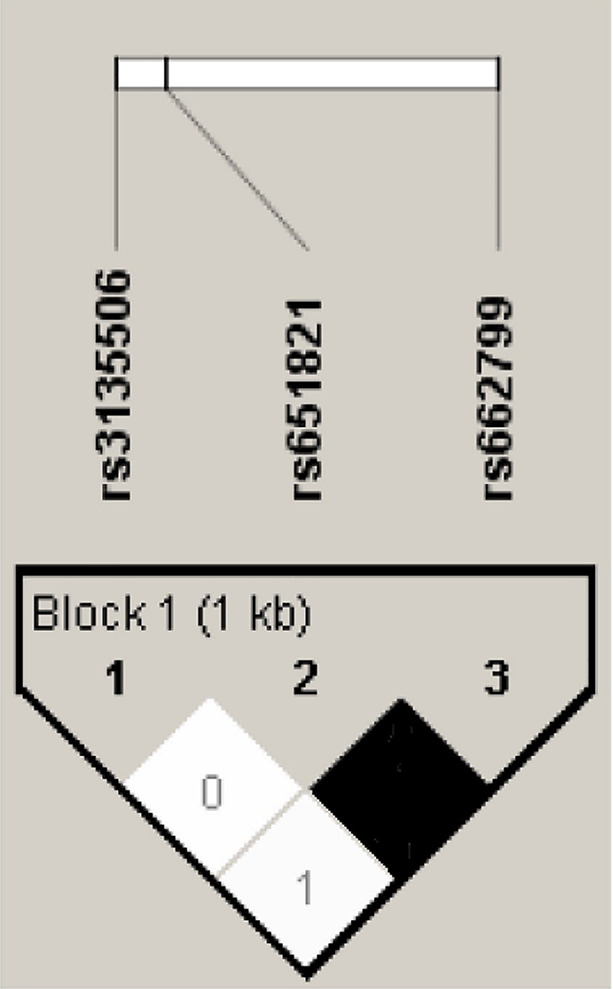
Linkage disequilibrium (LD) between the three single-nucleotide polymorphisms (*APOA5* SNPs) in Tunisian population The number in squares indicates the r^2^ index of LD between the correspondent SNPs.

We studied the linkage disequilibrium (LD) between the *APOA5* variants rs651821 and rs3135506 analyzed in our previous study [19] and the *APOA5* variant rs662799 analyzed in the present study. r2 index was estimated for all pairs of polymorphisms. The LD pattern showed a relatively high LD between SNPs rs651821 and rs662799 and a low LD between these two SNPs and rs3135506 (Figure 1). Haplotype analysis of the three *APOA5* SNPs (rs662799-rs3135506-rs651821) revealed three possible haplotypes. The major haplotype is the most common being AGT (77.7%), ACT (10.5%) and GGC (11.3%). Only the haplotype AGT showed a significant association with MetS by decreasing the risk of the disease OR = 0.659 (0.48-0.906) p = 0.009 (Table 6).

**Table 6:** Association analysis of haplotypes derived from genotype data

Haplotypes	rs662799	rs3135506	rs651821	Total frequency	Frequency		OR	Padj
					MetS	controls		
H1	G	G	C	0.113	0.127	0.093	1.498 (0.995-2.25)	0.051
H2	A	C	T	0.105	0.113	0.101	1.3 (0.834-2.03)	0.246
H3	A	G	T	0.777	0.76	0.805	0.659 (0.48-0.906)	0.009*

* p-value after Bonferroni correction. Significant results are in bold; Padj: p-value corrected for Age, sex and BMI.

### Meta-analysis

Meta-analysis was performed using available association data of *APOA5* gene with MetS in North African populations. Only two studies were found in addition to the present study [15,23]. The investigated common data are related to two genotyped *APOA5* gene variants: rs662799 and rs3135506 in the Tunisian and the Moroccan populations. We pooled the overall effect of rs662799 and rs3135506 on MetS risk for 594 Tunisians (299 controls and 295 patients) and 281 Moroccan (105 controls and 176 patients) [19,20] (Table 7). The pooled OR showed a significant association of the SNP rs3135506 (OR = 1.41, p = 0.015, *P_Heterogeneity_*_=_ 0.16 and I^2^ = 49.57%) and the SNP rs662799 (OR = 1.75, p<10^−3^; *P_Heterogeneity_*_=_ 0.12 and I^2^ = 57.97% with MetS (fixed effect).

**Table 7:** Meta-analysis of the North African region

SNP	A1	A2	P	OR	Q	I²
rs3135506	C	G	0.01595	1.4084	0.1591	49.57
rs662799	G	A	3.873e-005	1.7532	0.1230	57.97

A1; A2: allele 1 and allele 2, P:p-value; Q:P-heterogeneity, I²:I∧2 heterogeneity index

## Discussion

*APOA5* has been implicated in the development of MetS in many studies [15,19,21]. According to the last report brought by the World Health Organization MetS among other non-communicable diseases would be the leading cause of deaths internationally [29]. The association of the *APOA5* variants rs651821 and rs3135506 with MetS in the Tunisian population have been published by our team [19]. We reported a significant association of rs651821 with MetS and the correlation of rs3135506 with the increase of TG levels. In this present study we assessed the association of the *APOA5* gene variant rs662799 with MetS and its components in the Tunisian population.

Our results showed an association of rs662799 with MetS under the additive OR = 1.55(1.04-2.31); p = 0.028 and dominant models OR = 1.77(1.10-2.86); p = 0.018 conveying a higher risk of developing this syndrome to the carrier of the minor allele C of this SNP. Our findings are in accordance with previous studies performed across the continents [20,21,30,31].The rs662799 was significantly associated with MetS in both codominant model (OR = 10.13 (4.65-22.06); p< 0.0001) and Dominant model (OR = 7.82; (3.79-16.14);p < 0.0001) in Moroccan population [20]. Rs662799 was strongly associated with MetS OR = 3.622 (1.2-10.936) p = 0.02 for the Hungarian population [21]. The minor C allele of this SNP was associated with an approximately 50% higher risk of MetS in Chinese population [30]; and for the Greek population a strong correlation was also found OR = 3.514 (1.065-11.585) p = 0.035 [31]. In addition a metanalysis study performed with 13863 individuals from Europe, Asia, and Latin America reported that the carrier of the rs662799 C allele had a 33% increased risk of developing MetS [32]. However other studies reported no correlation of rs662799 with MetS [33,34].

The rs662799 is located in the promoter region that influences the expression levels of *APOA5* gene. Carriers of the minor allele C of this SNP present 20 to 30% higher plasma TG levels compared to individuals homozygous for the major allele T [35]. Kim et al, observed a decrease of HDL levels resulting in an increase of TG levels among carriers of the minor allele C of rs662799 [36]. They suggested that this SNP might modulate *APOA5* expression at the post-transcriptional level causing liver post-transcriptional down-regulation of *APOA5* by miR-485-5p. This hypothesis accounts, at least partially, for the subsequent elevation of plasma TG levels which warrants further experimental confirmation [37]. Thus, further investigations on the role of rs662799 in the gene expression, need to be conducted in particular in relation with dietary fat intake and obesity. These studies may help understand the impact of *APOA5* variants's dynamic changes on the lipid pathway and the risk of developing MetS.

Regarding the investigation of the sex impact on the genotype distribution of rs662799, our results showed an association with MetS only for women under the additive OR = 1.60(1.02-2.52); p = 0.037 and dominant OR = 2.00(1.13-3.54); p = 0.016 models. Gender-specific association of genetic variants with MetSwas also reported in otherprevious studies [19,38]. The SNP rs651821 in *APOA5* was positively associated with MetS in the women subgroup under the additive model OR = 1.63(1.08-2.59), p = 0.035 and under the dominant model OR = 2.11(1.18-3.77), p = 0.01 [19]. Elouej et al reported the association of rs1562398 in *LRPAP1* with MetS among the women group (p = 0.021) [38].

The variant rs662799 has been linked to other health problem including MetS traits. In studies performed on Iranian and Japanese populations, an association of this variant was reported with hypertension and the modulation of blood pressure [13,39]. In Puerto Rican study, the rs662799 was correlated with TG levels and total cholesterol [34]. However, we did not find the same results for our studied population. Association analysis of rs662799 with MetS quantitative traits such as fasting plasma glucose, BMI, LDL, HDL, systolic blood pressure, diastolic blood pressure, triglycerides and waist circumference showed the absence of any significant association. Our findings are still in accordance with others studies showing no correlation between the rs662799 and HDL levels [40], BMI [41] and TC levels [42].

In addition, we performed in this study an assessment of the cardiovascular risk among our MetS patients versus controls. Since, several studies reported a positive association between rs662799 and different cardiovascular problems such as coronary heart disease, ischemic stroke, coronary artery disease, arterial stiffness and myocardial infarction in various populations (Moroccan [11], Chinese [12], Japanese [13] and Italian population [14]).

Our results showed the absence of a significant correlation between rs662799 and cardiovascular risk (data not shown). Our finding is in accordance with others previous studies reporting a lack of association between rs662799 and cardiac diseases in the Canadian population [43], Pakistani population [44], Costa Rican [21] and the United Kingdom population [45].

Discrepancies between association studies can be explained by (i) the heterogeneity of clinical features for multifactorial diseases (MetS and heart problems) and the variability of their prevalence in different populations. (ii) The multitude of criteria and definitions used for phenotype characterization of patients (gender, age and different definitions for MetS: IDF 2005,NCEP ATP III etc.) and (iii) The interethnic variations of the allele distribution; according to ALFA project the risk allele frequency of rs662799 for European is 6.4%, African 12.5%, African American 12.6%, Asian 39%, East Asian 38%, south Asian 10%, Latin American group 1, 10.1% and Latin American group 2 17% [46].

In order to minimize discordances between association studies, it is important to standardize their design (gender, age range, clinical features ect.). Even though with these precautions we cannot control heterogeneity related to different genetic background. Consequently, it is important to perform specific association study for different populations.

Regarding the impact of the geographic origin on the genotype distribution of rs662799, our results showed a significant association with MetS for individuals originated from the North of Tunisia under additive and dominant models. Hence, the Northern population is distinctively prone to be more affected with MetS. The same result was observed for other SNP located in *APOA5* gene reported in our previous study [19]. This result highlights a variability between regions underling a mosaic genetic landscape of the Tunisian population [28,47].

In the present study we performed also a haplotype analysis to assess the contribution of *APOA5* haplotypes in MetS development. We observed that the haplotype AGT which is a combination of rs662799-rs3135506-rs651821 major alleles, is significantly associated with MetS OR = 0.659 (0.48-0.906); p = 0.009. This haplotype confers a protective effect against MetS. Other studies have reported association of *APOA5* haplotypes with either MetS or one of its traits [48–50].

In the second part of our work we performed a meta-analysis of the association of *APOA5* variants with MetS in North Africa. According to the literature only the Tunisian and Moroccan populations were investigated. The common genotyped variants in *APOA5* are rs662799 (present study) and rs3135506 [20].

Our results showed a significant association of the variants rs662799 and rs3135506 with MetS. The variants rs662799 and rs3135506 increase the risk of MetS by 1.75-fold (p<10^−3^) and 1.41-fold (p = 0.015) respectively. Our findings are consistent with several other meta-analyses performed in ethnic groups where the *APOA5* variants were considered to be a strongly implicated in the development of MetS globally. A meta-analysis performed after pooling 12 studies including 5,573 MetS cases and 8,290 controls from Asia and Europe showed that rs662799 was associated with increased risk of MetS with an OR  =  1.33 (1.16, 1.53) in the overall population, OR = 1.43 (1.29, 1.58) in East Asian and OR = 1.30 (0.94, 1.78) in European populations [32]. Another meta-analysis study showed that C allele carriers (CC + TC) of rs662799 had a significantly higher risk of MetS for the overall (OR = 1.32 (1.14-1.53) *P*=0.000) [51].

The present case-control study, pinpoints the major role of *APOA5* gene in the pathogenesis of MetS for the Tunisian population; we demonstrated the correlation of rs662799 variant with MetS. We highlighted also an inter-regional difference and a gender-specific association of this variant with MetS. However, no associations were found of the SNP rs662799 with the components of MetS. We showed that AGT haplotype integrating major alleles of rs662799-rs3135506-rs651821, is negatively correlated with MetS conferring a protective effect for the carriers of this haplotype. This is the first meta-analysis conducted in the North African region, showing the implication of the *APOA5* variants (rs662799 and rs3135506) in the increasing risk of developing MetS. Further explorations and meta-analysis studies encompassing other countries and a larger sample size, need to be done in order to further showcase the important role of *APOA5* gene in MetS development.

## Abbreviations

95% CI: 95% confidence interval
AIC: Akaike Information Criterion
ALFA: Allele Frequency Aggregator
ANOVA: Analysis of Variance
*APOA5*: apoliprotein A5
BMI: body mass index
D': coefficient of linkage disequilibrium
DB: database
DBP: diastolic blood pressure
DT2: Type 2 diabetes
GWAS: genome-wide association study
HDL: high-density lipoprotein
HNF4A: hepto nuclear factor 4α
HWE: Hardy–Weinberg equilibrium
*I^2^*: heterogeneity index
IFG: impaired fasting glucose
IPT: Institut Pasteur de Tunis
KASPar: Kompetitive Allele Specific PCR
LD: linkage disequilibrium
LDL: low-density lipoprotein
LPL: lipoprotein lipase
MetS: metabolic syndrome
N: number
NIN: National Institute of Nutrition
OR: odds ratio
*P*: *P*-value
*P*_adj_: *P*-value adjusted
Q: *P*-value heterogeneity
r^2^: correlation coefficient
RAF: risk allele frequency
SBP: systolic blood pressure
SD: standard deviation
SNP: single nucleotide polymorphism
TC: total cholesterol
TG: triglyceride
VLDL-TG: very low-density lipoprotein-triglyceride
WC: Waist Circumference
X2: Chi-squared test
